# Identification of *Streptococcus pneumoniae* by a real-time PCR assay targeting SP2020

**DOI:** 10.1038/s41598-019-39791-1

**Published:** 2019-03-01

**Authors:** Débora A. Tavares, Sara Handem, Ricardo J. Carvalho, A. Cristina Paulo, Hermínia de Lencastre, Jason Hinds, Raquel Sá-Leão

**Affiliations:** 1 Laboratory of Molecular Microbiology of Human Pathogens, Instituto de Tecnologia Química e Biológica António Xavier, Universidade Nova de Lisboa (ITQB-NOVA), Oeiras, Portugal; 2 Laboratory of Molecular Genetics, ITQB-NOVA, Oeiras, Portugal; 3 Laboratory of Microbiology and Infectious Diseases, The Rockefeller University, New York, NY USA; 4 Institute for Infection and Immunity, St George’s University of London, London, UK; 5 Departamento de Biologia Vegetal, Faculdade de Ciências, Universidade de Lisboa, Lisboa, Portugal

## Abstract

Real-time PCR targeting *lytA* (the major autolysin gene) and *piaB* (permease gene of the *pia* ABC transporter) are currently used as the gold-standard culture-independent assays for *Streptococcus pneumoniae* identification. We evaluated the performance of a new real-time PCR assay – targeting SP2020 (putative transcriptional regulator gene) – and compared its performance with the assays previously described. A collection of 150 pneumococci, 433 non-pneumococci and 240 polymicrobial samples (obtained from nasopharynx, oropharynx, and saliva; 80 from each site) was tested. SP2020 and *lytA*-CDC assays had the best performance (sensitivity of 100% for each compared to 95.3% for *piaB*). The specificity for *lytA* and *piaB* was 99.5% and for SP2020 was 99.8%. Misidentifications occurred for the three genes: *lytA*, *piaB* and SP2020 were found in non-pneumococcal strains; *piaB* was absent in some pneumococci including a serotype 6B strain. Combining *lytA* and SP2020 assays resulted in no misidentifications. Most polymicrobial samples (88.8%) yielded concordant results for the three molecular targets. The remaining samples seemed to contain non-typeable pneumococci (0.8%), and non-pneumococci positive for *lytA* (1.7%) or SP2020 (8.7%). We propose that combined detection of both *lytA*-CDC and SP2020 is a powerful strategy for the identification of pneumococcus either in pure cultures or in polymicrobial samples.

## Introduction

Identification of the human pathogen *Streptococcus pneumoniae* (or pneumococcus) is an important task that may pose challenges. For example, in pneumococcal carriage studies investigating vaccine impact and resistance to antibiotics, it was found that some isolates were prone to be misidentified resulting in over-estimation of rates of antimicrobial resistance^1–3^.

The WHO recommended algorithm for routine identification of pneumococcus relies on optochin susceptibility, bile solubility, and serotyping of cultured α-hemolytic colonies. Nevertheless, atypical results to these traditional phenotypic assays have been described^4–6^. Molecular assays have been used as an alternative but were found to be hampered by the frequent genetic exchange between pneumococcus and other streptococcus of the viridans group, mainly *S. pseudopneumoniae* and *S. mitis*^6^.

For culture-independent assays the current method of choice is a real-time PCR assay targeting the gene *lytA* (*lytA*-CDC)^7,8^. LytA is the major autolysin of pneumococcus and has been described as ubiquitous and specific of this species^9^. The performance of this real-time PCR assay was initially tested with a collection of 67*S. pneumoniae* and 104 non-pneumococcal isolates. The latter group included 13 viridans group streptococci not identified to the species level. This method has been extensively used by different laboratories in both disease and carriage studies^10,11^.

A second real-time PCR assay, targeting *piaB*, a permease of an ABC transporter involved in iron uptake and virulence, has also been used to increase the specificity of pneumococcal identification^11–13^. Although this system has been described as pneumococcus-specific, it is not ubiquitous, being absent from some non-encapsulated pneumococci (non-typeable, NT)^14,15^. Proper identification of NTs is of relevance as they are frequently multiresistant to antibiotics, preferential hubs for horizontal gene transfer and their prevalence in carriage is significant and appears to be increasing since the introduction of pneumococcal conjugate vaccines (PCVs)^16–19^. From a clinical perspective NT have been frequently associated with conjunctivitis outbreaks^20–22^.

Albeit there has been some evidence that homologues of *lytA* (and of other pneumococcal genes) can be present in closely related species of *Streptococcus*, until recently, this had not been sufficiently tested^6,23,24^: a study from Wyllie *et al*., published in 2017, which included hundreds of streptococcal isolates suggested that the *lytA*-CDC and *piaB* real-time PCR assays most frequently in use are 100% specific for *S. pneumoniae*^7,11,25^.

Apart from *lytA* and *piaB*, other pneumococcal genes such as the ones encoding for pneumolysin (*ply*) or the pneumococcal surface adhesin A (*psaA*) have also been tested as targets for the identification of pneumococcus. However, these were abandoned due to poorer specificity when compared to that of *lytA*^7,25^.

Other methods have also been proposed such as determination of pneumococcal-specific sequence signatures for 16S rRNA^26^ or S2 ribosomal protein^25^, and the identification of pneumococcus by MALDI-TOF^27–29^. A potential disadvantage of these methods is that they are best suited for testing pure cultures that may not always be available, for example, when pneumococcal carriage is being detected from polymicrobial samples.

One possible candidate for accurate pneumococcal identification is SP2020. SP2020 is a putative transcriptional regulator of the GntR-family and belongs to the core genome of pneumococcus^30,31^. Very recently, while we were in the process of publishing our study, Croxen *et al*. described SP2020 as a good marker to discriminate between *S. pneumoniae* and *S. pseudopneumoniae*^32^. The authors performed an *in silico* analysis based on published complete genomes which suggested that SP2020 was nearly universally present in pneumococci and absent from all non-pneumococcal streptococci. The authors also designed a real-time PCR assay targeting this marker, and tested it on a panel of 36 pneumococci and 149 non-pneumococcal streptococcal isolates^32^.

In this study we aimed to evaluate the performance of a new real-time PCR assay targeting SP2020 and to compare its performance with the *lytA*-CDC and *piaB* assays previously described. For that, a large collection of α-hemolytic non-pneumococcal isolates (n = 402) was tested as well as two control collections: one included 150 pneumococcal strains (of 50 serotypes and NTs); the other consisted of 31 strains of 23 non-pneumococcal *Streptococcus* species. Finally, the assays were tested against a collection of polymicrobial samples obtained from the nasopharynx (n = 80), oropharynx (n = 80) and saliva (n = 80).

## Results and Discussion

### Evaluation of *lytA, piaB*, and SP2020 real-time PCR assays in pure cultures of pneumococci

**Figure 1:**
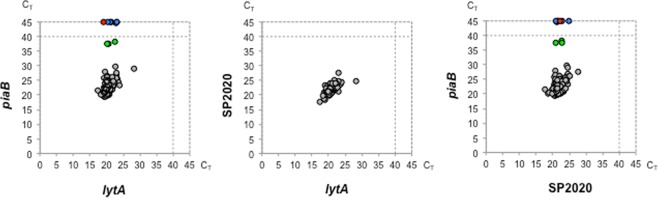
C_T_ values obtained for 150 *S. pneumoniae* tested by real-time PCR for the presence of *lytA*, *piaB* and SP2020. Six NT strains (blue circles) and one 6B strain (red circle) had no amplification for *piaB*. Three NT strains (green circles) yielded C_T_ values for *piaB* far higher than those obtained for *lytA* and SP2020.

To evaluate the sensitivity of real-time PCR assays targeting *lytA*, *piaB*, and SP2020, 150 pneumococcal strains were used. For all pneumococcal strains, a positive real-time PCR result was obtained for *lytA*, and SP2020; in addition, 143 (95.3%) strains were positive for *piaB* (Fig. 1). Hence, by using this collection, the sensitivity of the *lytA*, and SP2020 assays was 100%, whereas that of *piaB* was 95.3% (Table 1).

**Table 1:** Sensitivity, specificity, positive predictive value (PPV), negative predictive value (NPV), and species misidentified by the real-time PCR assays tested.

Assay (C_T_ ≤ 40)	Sensitivity	Specificity	PPV	NPV	Misidentified species (no. isolates out of 577)
*lytA*	100%	99.5%	98.7%	100%	*S. pseudopneumoniae* (2)
*piaB*	95.3%	99.5%	98.6%	98.4%	*S. pneumoniae* (7), *S. pseudopneumoniae* (2)
SP2020	100%	99.8%	99.3%	100%	*S.mitis-S.pseudopneumoniae* group (1)
*lytA*+*piaB*	95.3%	100%	100%	98.4%	*S. pneumoniae* (7)
*lytA*+SP2020	100%	100%	100%	100%	—
*piaB*+SP2020	95.3%	100%	100%	98.4%	*S. pneumoniae* (7)
*lytA*+*piaB*+SP2020	95.3%	100%	100%	98.4%	*S. pneumoniae* (7)

The seven pneumococcal strains that had a negative result for *piaB* were of capsular types NT (n = 6) and 6B (n = 1) (Fig. 1, Table 2).

**Table 2:** Properties of non-typeable *S. pneumoniae* and other strains with unusual results when tested by real-time PCR for *lytA*, *piaB*, and SP2020.

Strain	Species classification^a^ (serotype)	Real-time PCR (C_T_)^b^			MLST allelic profile^c^							ST
		*lytA*	*piaB*	SP2020	*aroE*	*ddl*	*gdh*	*gki*	*recP*	*spi*	*xpt*	
DCC1365	*S. pneumoniae* (NT)	21	37	21	8	53	37	9	29	2	12	344
DCC635	*S. pneumoniae* (NT)	23	NA	23	8	53	37	9	29	2	12	344
DCC646	*S. pneumoniae* (NT)	23	NA	21	8	53	37	9	29	2	12	344
PT526b	*S. pneumoniae* (NT)	23	39	23	8	53	37	9	29	2	12	344
PT1493	*S. pneumoniae* (NT)	20	24	21	2	164	13	2	29	91	19	1617
PT1683	*S. pneumoniae* (NT)	21	22	21	7	1	11	10	1	6	8	156
PT1718	*S. pneumoniae* (NT)	22	NA	22	70	59	10	15	16	2	105	1540
PT1804b	*S. pneumoniae* (NT)	22	NA	22	8	26	74	19	15	6	40	888
PT2293b	*S. pneumoniae* (NT)	21	NA	21	8	53	37	9	29	2	12	344
PT3201	*S. pneumoniae* (NT)	21	26	22	2	141	13	2	29	91	19	1153
PT4014	*S. pneumoniae* (NT)	20	26	22	2	141	13	2	29	91	19	1153
PT4222	*S. pneumoniae* (NT)	20	26	21	2	59	13	2	29	91	19	1156
WL1084	*S. pneumoniae* (NT)	21	37	23	8	71	5	2	27	2	11	448
WL1514	*S. pneumoniae* (NT)	23	NA	25	8	53	37	9	29	2	12	344
ATCC BAA-342	*S. pneumoniae* (6B)	20	NA	22	7	67	6	9	2	6	1	384
EL2652N1	*S. pseudopneumoniae*	NA	24	NA	103	656	477 (98)	250 (97)	264 (97)	313 (99)	595 (95)	—
Spain939	*S. pseudopneumoniae*	NA	26	NA	427	751 (97)	492	345 (99)	139 (98)	442 (99)	105 (99)	—
Spain2270	*S. pseudopneumoniae*	24	NA	NA	427	447 (97)	381 (98)	197 (94)	373	442 (99)	735 (96)	—
Spain9880	*S. pseudopneumoniae*	24	NA	NA	427	447 (97)	381 (98)	197 (94)	373	442 (99)	735 (96)	—
Spain3473	*S. pseudopneumoniae/S.mitis*	NA	NA	23	427	751 (97)	40	382 (99)	29	442 (99)	105 (99)	—

^a^Species classification was done based on MLST/MLSA as described in Materials and Methods section. ^b^NA, no amplification in 45 cycles. ^c^MLST allelic profile of non-pneumococcal strains indicates the allele number of the closest match; the similarity (in %) is indicated in parenthesis. *S. pneumoniae* MLST database was last accessed on February 8, 2018.

The absence of *piaB* has been previously reported for NT pneumococci^14,15^. Among the 14 NT isolates tested only five had C_T_ values for *piaB* that were concordant with those obtained for *lytA* and SP2020. Three other NTs yielded C_T_ values for *piaB* <40 but far higher than those obtained for *lytA* and SP2020; six did not have any amplification for *piaB* in 45 cycles (Fig. 1, Table 2). All but one strain were true NTs, i.e. they were non-encapsulated belonging to *cps* type NCC2a or NCC2b^14^. The other strain was a non-encapsulated derivative of a capsulated ST156 lineage (strain PT1683)^16^.

**Figure 2:**
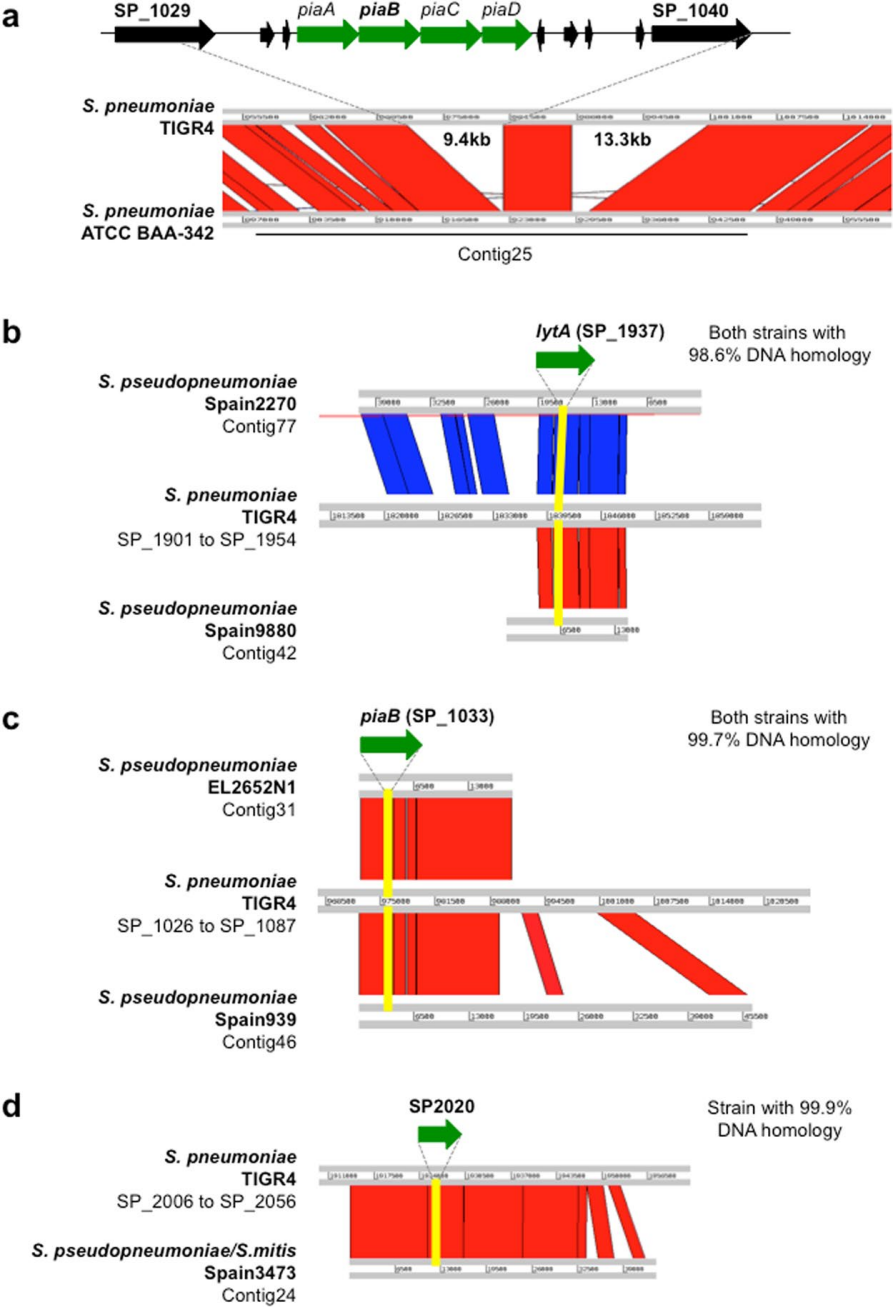
Genomic comparison between regions of interest in TIGR4 (NCBI accession number AE 005672.3) and strains (pneumococci and non-pneumococci) with atypical results when tested for the presence of *lytA*, *piaB*, or SP2020: (**a**) serotype 6B pneumococcal strain (ATCC BAA-342) testing negative for *piaB*; **(b)** *S. pseudopneumoniae* strains Spain2270 and Spain9880 testing positive for *lytA*; (**c**) *S. pseudopneumoniae* strains EL2652N1 and Spain939 testing positive for *piaB*; **(d)** *S. pseudopneumoniae/S. mitis* strain Spain3473 testing positive for SP2020. Regions with nucleotide identity ≥93% are represented either in red (same orientation) or in blue (reverse orientation). Regions highlighted in yellow indicate the gene of interest. All comparisons were performed by Double ACT v2 and visualized using Artemis Comparison Tool (ACT) release 17.0.1.

We also identified a serotype 6B isolate lacking *piaB*. To our best knowledge, the absence of the *pia* locus in capsulated pneumococci has not been described before. Whole genome sequencing of this strain (strain ATCC BAA-342, the prototype of Maryland^6B^-17 PMEN clone)^33^ confirmed the presence of the 6B capsular locus, the MLST 384 profile, and the lack of *piaB*. Comparison with the TIGR4 genome revealed the entire *piaAD* locus (3.8 kb) as well as an adjacent region of 5.6 kb was absent from ATCC BAA-342 (Fig. 2a). Since serotype 6B is targeted by pneumococcal conjugate vaccines, misidentification of pneumococcus based on *piaB* detection could potentially affect studies aiming to evaluate vaccine efficacy.

### Evaluation of *lytA, piaB*, and SP2020 real-time PCR assays in pure cultures of non-pneumococcal streptococci

**Figure 3:**
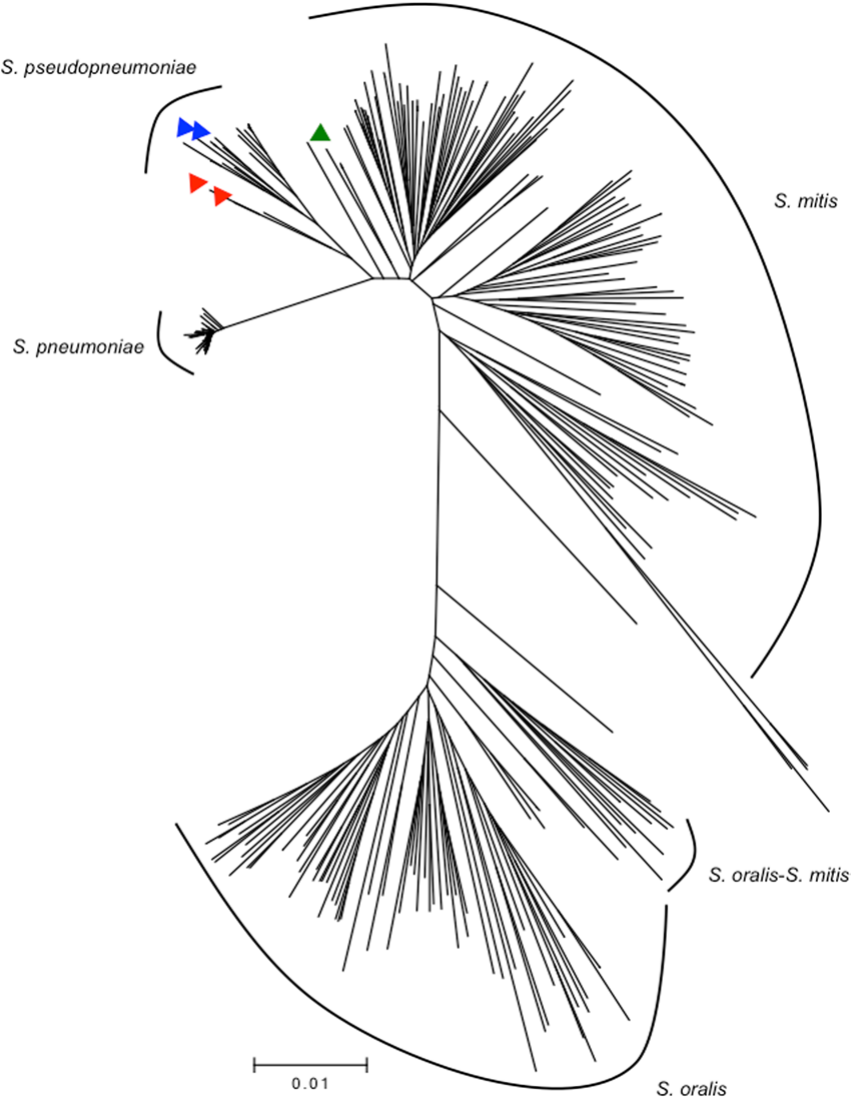
Phylogenetic tree based on concatenated MLSA sequences of the strains analyzed in this study and *S. pneumoniae*, *S. pseudopneumoniae*, *S. mitis*, and *S. oralis* strains deposited at the eMLSA database. Five non-pneumococcal isolates giving a positive result for at least one of the real-time PCR assays were tested. Triangles indicate strains analyzed in this study: red – *lytA*^+^, blue – *piaB*^+^, green – SP2020^+^.

To evaluate the specificity of the real-time PCR assays, 433 non-pneumococcal streptococcal isolates were tested. In total 98.8% of the isolates (n = 428) gave a negative result for *lytA*, *piaB*, and SP2020. Five isolates were positive for one of the assays (Table 2). Whole genome sequencing was done for these five isolates to confirm the presence of the regions of interest (containing *lytA*, *piaB* or SP2020) and for species identification based on MLST and MLSA. None of the five isolates was *S. pneumoniae* (Table 2, Fig. 3). Four isolates were identified as *S. pseudopneumoniae*: two contained *lytA* and two contained *piaB* (Fig. 2b,c). One isolate was positive for SP2020 and could not be speciated as it fell in between *S. pseudopneumoniae* and *S. mitis* (Fig. 2d).

The identification of SP2020 in a non-pneumococcal strain is novel and contrasts with the study of Croxen *et al*., where SP2020 was universally absent from 3,405 non-pneumococcal streptococcal genomes and 46,727 non-streptococcal genomes (viral, fungal and bacterial genomes associated with sputum and nasopharyngeal samples) publicly available at the time^32^.

Based on these results the specificity for *lytA* and *piaB* was 99.5% and for SP2020 was 99.8% (Table 1).

### Performance of *lytA*, *piaB*, and SP2020 in pure cultures

Globally, when the results obtained for pneumococci and streptococci of other species were combined, the positive predictive values (PPV) of the real-time PCR assays were 98.7% for *lytA*, 98.6% for *piaB* and 99.3% for SP2020. The negative predictive values (NPV) were 100% for *lytA* and SP2020, and 98.4% for *piaB* (Table 1).

The assays with best performance for the identification of pneumococcus in pure samples were SP2020 and *lytA*-CDC (Table 1, Supplementary Table 1). Of importance, combining *lytA* and SP2020 assays resulted in no misidentifications: all pneumococci tested contained both genes and, among the other streptococci no strain was found carrying simultaneously the two genes (Table 1).

### *In silico* screening for *lytA, piaB*, and SP2020 of pneumococcal genomes

To complement our observations, we performed an *in silico* analyses to screen for the absence of *lytA, piaB*, or SP2020 in pneumococcal genomes publicly available (https://www.ncbi.nlm.nih.gov/genome/?term=streptococcus+pneumoniae). Among 8,251 pneumococcal genomes available at NCBI database (accessed July 9, 2018), *lytA* was absent from one genome (0.01%, serotype 3), *piaB* was absent from 250 genomes (3.0%, NTs and capsulated strains of 12 different serotypes), and SP2020 was absent from 23 genomes (0.28%, NTs and capsulated strains of 4 serotypes). One genome lacked both *piaB* and SP2020 (Supplementary Table 2). The SP2020 region targeted by the real-time PCR assay described in this study was 100% identical in 8,181 genomes and over 99% identical in 40 genomes. For the remaining six genomes this region was incomplete but 100% identical.

### Evaluation of *lytA*, *piaB*, and SP2020 real-time PCR assays in polymicrobial samples

**Figure 4:**
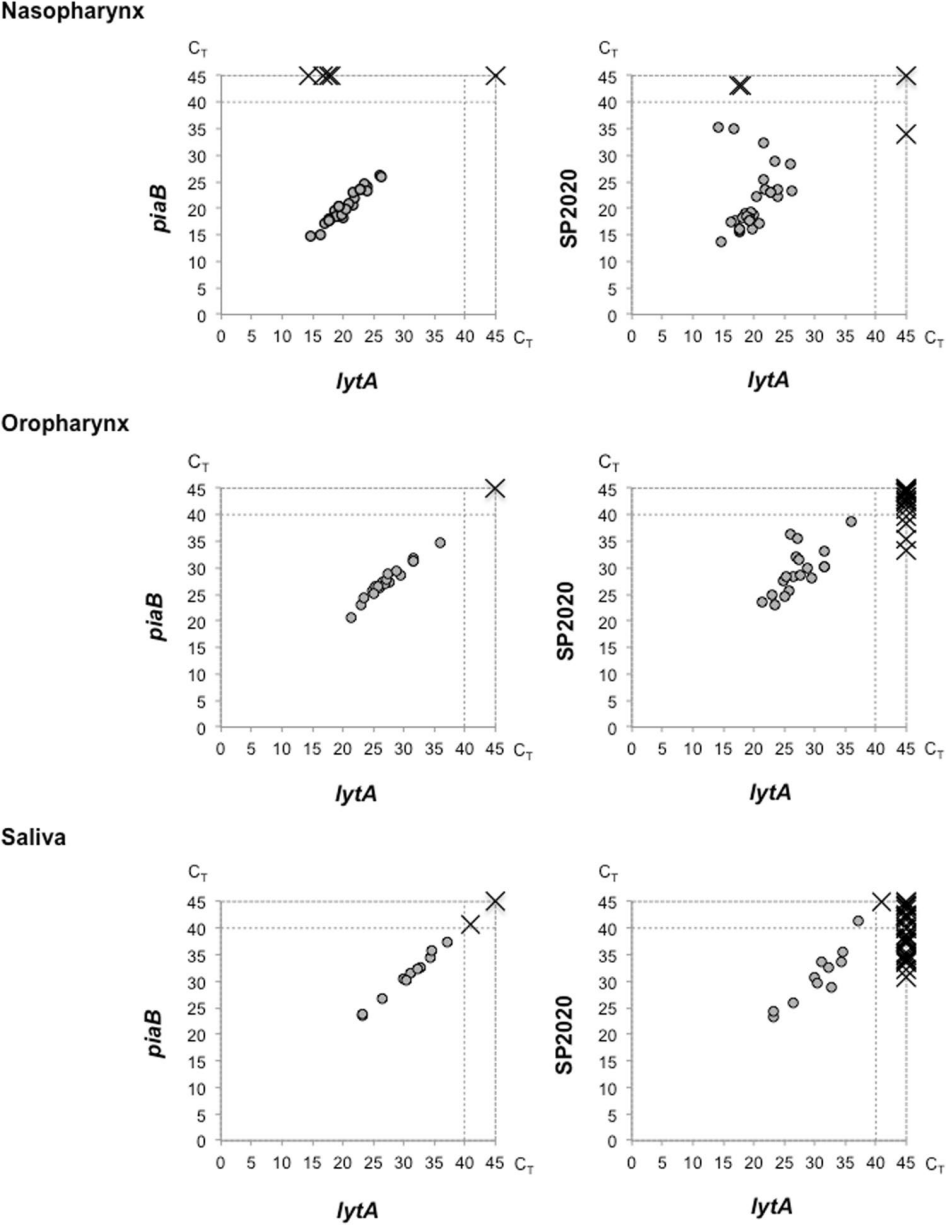
C_T_ values obtained for 240 polymicrobial samples tested by real-time PCR for the presence of *lytA*, *piaB* and SP2020. Samples obtained from the nasopharynx, oropharynx and saliva of healthy adults (80 each) were tested. Circles indicate samples found to contain pneumococci (based on real-time PCR results for *lytA*, *piaB*, and SP2020 and real-time PCR capsular assignment). Crosses indicate samples for which no pneumococcus was detected. Additional details on these results are provided in Table 3.

The three real-time PCR assays were tested in 240 polymicrobial samples obtained from the nasopharynx, oropharynx and saliva of adults (n = 80 for each type of sample). Most samples (88.8% of 240) yielded concordant results (either positive or negative and with comparable C_T_ values) for the three molecular targets (Table 3, Fig. 4, Supplementary Table 3). Of notice, five samples (three from the nasopharynx and two from the oropharynx) yielded C_T_ values for SP2020 <40 but far higher than those obtained for *lytA* and *piaB* (Fig. 4, Supplementary Table 3).

**Table 3:** Real-time PCR results for polymicrobial samples obtained from nasopharynx, oropharynx and saliva.

Positive real-time PCR assay^a^	Nasopharynx (n = 80)	Oropharynx (n = 80)	Saliva (n = 80)	Observations
*lytA*, *piaB*, SP2020	25 (31.2%)	18 (22.5%)	11 (13.8%)^b^	capsulated pneumococci^c^
*lytA*, SP2020	2 (2.5%)	0	0	NT pneumococci^d^
*lytA*	4 (5.0%)	0	0	*S. pseudopneumoniae* ^e^
SP2020	1 (1.3%)	4 (5.0%)	16 (20.0%)	*S. pseudopneumoniae/S.mitis* ^f^
Negative for all	48 (60.0%)	58 (72.5%)	53 (66.2%)	—

^a^Three targets were tested: *lytA, piaB* and SP2020. ^b^Includes one sample with borderline C_T_ values (37 for *lytA* and *piaB* and 41 for SP2020) for which the capsular type assigned by real-time PCR was 7A/7F suggesting the presence of pneumococci at low density. ^c^Serotyping by real-time PCR led to assignment of capsular type/group to all samples. Nasopharyngeal samples: serotypes 3 (n = 1), 6C (n = 4), 8 (n = 2), 10A (n = 2), 15C (n = 1), 19A (n = 2), 23A (n = 2), 23B (n = 1), 35F (n = 1), 37 (n = 7), NT (n = 2). Oropharyngeal samples: serotypes 3 (n = 1), 6C (n = 1), 8 (n = 2), 10A (n = 2), 19A (n = 3), 22A/F (n = 1), 23A (n = 1), 35B (n = 1), 35F (n=2), 37 (n = 4). Saliva samples: serotypes 6C (n = 1), 7A/F (n=2), 7A/F and 15B/C (n = 1), 8 (n = 2), 11A/D (n = 3), 35F (n = 1), 37 (n = 1). ^d^For one of the samples a pneumococcal strain was isolated in pure culture and confirmed to be NT. For the other sample a NT-specific PCR was done as described in the Materials and Methods section. ^e^Species assignment was done based on the results obtained when the assays were validated using the collections of isolates (150 pneumococci and 433 other streptococci) described in the Materials and Methods section.

As no discrepancies were observed in the efficiency of the assays when pure cultures were tested, it seems unlikely that this would have happened in these five specific samples. Whether the large difference in C_T_ values was due to the presence of mixed strains lacking at least one of the targets remains unclear as the specific strains yielding these results were not isolated from the polymicrobial samples (see below).

The presence of pneumococci was detected in 31.2%, 22.5% and 13.8% of the nasopharyngeal, oropharyngeal and saliva samples, respectively. For all these samples a capsular type was assigned based on real-time PCR serotyping (Table 3). The presence of NT pneumococci was detected in two additional nasopharyngeal samples that were negative for *piaB* (Table 3, Fig. 4, Supplementary Table 3).

A total of 25 samples tested positive for only one molecular target: 4 were positive for *lytA* and 21 were positive for SP2020. The *lytA* positive samples were all obtained from the nasopharynx (4 out of 80, 5.0%), and possibly contained *S. pseudopneumoniae*. The SP2020 positive samples were obtained from the nasopharynx (1 out of 80, 1.3%), the oropharynx (4 out of 80, 5.0%), and saliva (16 out of 80, 20.0%) suggesting the presence of *S. pseudopneumoniae/S. mitis* (Table 3, Fig. 4, Supplementary Table 3).

In an attempt to isolate from polymicrobial samples pure cultures positive for SP2020 only, the saliva sample with the lowest C_T_ (C_T_ of 31) was serially diluted and cultured to obtain isolated colonies. Six-hundred colonies were picked and tested by real-time PCR assays for the presence of SP2020 and *lytA*. All 600 colonies were negative for both assays suggesting that the bacteria that contributed to positive assays were present at low density.

Taken together, the analysis of polymicrobial samples from the nasopharynx, oropharynx and saliva suggest that, as samples become increasingly complex, the chance of detecting positive signals for individual “pneumococcal markers” increases. In the polymicrobial samples we tested, this was particularly relevant when the presence of SP2020 was tested in saliva samples.

The contrast between the results obtained for SP2020 when pure cultures were analyzed (0.23% false positives, n = 1) with the results obtained in saliva samples (20% false positives) suggests that our test collection of α-hemolytic non-pneumococcal isolates was biased. This collection was obtained from nasopharyngeal and oropharyngeal samples and appears not to be representative of the plethora of non-*S. pneumoniae Streptococcus* spp. present in saliva. Further studies aiming to understand streptococcal diversity in the human body would be of added value to improve molecular diagnostics of *S. pneumoniae*.

## Conclusions

The real-time PCR assay here described targeting SP2020 is useful for the correct identification of pneumococci when used in combination with *lytA*. Although the individual presence of these genes was detected in non-pneumococcal strains, no misidentifications occurred when both assays were combined. In particular, all NT pneumococci, which appear to be of increasing epidemiological relevance in carriage^17,19^, were correctly identified.

Analysis of polymicrobial samples unveiled the complexity and risks of inferring the presence of pneumococci using molecular methods only. These observations are in line with studies from Carvalho *et al*. and Wyllie *et al*. who identified homologs of pneumococcal serotype-specific gene sequences in non-pneumococcal mitis-group streptococci, confounding the detection of pneumococci in polymicrobial samples^25,34^. Their findings and our results, emphasize the importance of using more than one target for the identification of pneumococci.

In conclusion, combined use of real-time PCR targeting *lytA* and SP2020 is an improved alternative to the detection of *lytA* alone or in combination with *piaB* and is useful for detection of pneumococci in pure cultures and in polymicrobial samples.

## Methods

### Study collections

Four collections were tested: a *S. pneumoniae* control collection (n = 150), a non-*S. pneumoniae Streptocococcus spp*. control collection (n = 31), a test collection of α-hemolytic non-pneumococcal isolates (n = 402), and a test collection of polymicrobial samples collected from the nasopharynx (n = 80), oropharynx (n = 80) and saliva (n = 80).

The *S. pneumoniae* control collection included 150 pneumococcal strains belonging to 50 serotypes plus non-typeables (NT, n = 14). These strains were obtained from carriage studies previously described^35^. Strains were previously characterized based on phenotypic tests (colony morphology, optochin susceptibility and bile solubility), serotyping (PCR and/or Quellung reaction) and genotyping (PFGE and/or MLST). In addition, NT were also tested by a multiplex-PCR based strategy targeting *lytA, cpsA, aliB-like ORF2*, and *16SrDNA* genes followed by a restriction fragment length polymorphism assay to differentiate typical from atypical *lytA*^16,35^. The represented serotypes were 1 (n = 2), 3 (n = 4), 4 (n = 2), 5 (n = 1), 6A (n = 4), 6B (n = 8), 7A (n = 1), 7F (n = 2), 8 (n = 1), 9A (n = 2), 9L (n = 2), 9N (n = 2), 9V (n = 3), 10A (n = 4), 11A (n = 4), 12A (n = 1), 12B (n = 1), 12F (n = 2), 14 (n = 8), 15A (n = 3), 15B (n = 2), 15C (n = 2), 15F (n = 2), 16F (n = 4), 17 (n = 2), 18A (n = 2), 18B (n = 2), 18C (n=2), 18F (n = 2), 19A (n = 6), 19F (n = 7), 20 (n = 1), 21 (n = 2), 22F (n = 2), 23A (n = 2), 23B (n = 2), 23F (n = 9), 24B (n = 1), 24F (n = 2), 29 (n = 2), 31 (n = 2), 33B (n = 1), 33F (n = 2), 34 (n = 2), 35B (n=1), 35F (n = 4), 37 (n = 2), 38 (n = 6), 39 (n = 1), and 42 (n = 2). This collection included the prototype strains of 27 Pneumococcal Molecular Epidemiology Network (PMEN) clones (http://www.sph.emory.edu/PMEN/index.htm): Spain^23F^-1, Spain^6B^-2, Spain^9V^-3, Tennessee^23F^-4, Spain^14^-5, Hungary^19A^-6, South Africa^19A^-7, South Africa^6B^-8, England^14^-9, CSR^14^-10, CSR^19A^-11, Finland^6B^-12, South Africa^19A^-13, Taiwan^19F^-14, Taiwan^23F^-15, Poland^23F^-16, Maryland^6B^-17, Tennessee^14^-18, Colombia^5^-19, Poland^6B^-20, Portugal^19F^-21, Greece^6B^-22, North Carolina^6A^-23, Utah^35B^-24, Sweden^15A^-25, Colombia^23F^-26, and Portugal^6A^-41.

The non-*S. pneumoniae Streptococcus spp*. control collection included 20 type strains of the following species: *S. mitis* (DSM-12643), *S. oralis* (DSM-20627), *S. cristatus* (DSM-8249), *S. gordonii* (DSM-6777), *S. infantis* (DSM-12492), *S. parasanguinis* (DSM-6778), *S. peroris* (DSM-12493), *S. sanguinis* (DSM-20567), *S. sinensis* (DSM-14990), *S. anginosus subsp. anginosus* (DSM-20563), *S. constellatus subsp. constellatus* (NCTC11325), *S. intermedius* (NCTC11324), *S. salivarius subsp. salivarius* (DSM-20560), *S. vestibularis* (DSM-5636), *S. agalactiae* (DSM-6784), *S. canis* (DSM-20715), *S. dysgalactiae sub. dysgalactiae* (DSM-20662), *S. equi sub. zooepidemicus* (DSM-20727), *S. mutans* (DSM-20523) and *S. pyogenes* (DSM-20565). In addition, it also included 11 strains of the following species: *S. pseudopneumoniae* (PT5479 and IS7943)^2,24^, *S. oralis* (DSM-20066 and DSM-20395), *S. gordoni* (DSM-20568), *S. dysgalactiae sub. equisimilis* (DSM-6176), *S. equinus* (NCTC10389 and DSM-20480) and *Streptococcus spp*. (DSM20377, DSM20379 and DSM20392). DSM strains were obtained from the Leibniz Institute DSMZ-German Collection of Microorganisms and Cell Cultures (www.dsmz.de) and NCTC strains were obtained from the National Collection of Type Cultures of Public Health England (www.phe-culturecollections.org.uk).

The test collection of α-hemolytic non-pneumococcal isolates included 402 isolates, recovered from humans between 1991 and 2012 from different carriage and disease studies and all belong to our collection^2,36,37^. Isolates were initially isolated based on the observation of α-hemolysis and colony morphology suggestive of pneumococcus, but were found to be of other streptococcal species when a combination of methods was applied (optochin susceptibility, bile solubility, serotyping, and *lytA*-BsaI-RFLP)^2,38^. Of the 402 α-hemolytic non-pneumococcal isolates tested, 346 were resistant to optochin, 25 were susceptible to optochin but bile insoluble, and 31 were susceptible to optochin and bile soluble but could not be assigned to a serotype. These latter 31 isolates were confirmed not to be pneumococcus by a multiplex PCR scheme previously described and the identification of characteristic non-pneumococcal *lytA*-BsaAI-RFLP signatures^35,38^.

The test collection of polymicrobial samples (from the nasopharynx, oropharynx and saliva, 80 each) was obtained between 2015 and 2016 from healthy adults aged 25 to 50 years old. Nasopharyngeal and oropharyngeal swabs were placed in STGG; saliva was collected by spitting into a tube and 1 ml was transferred to a tube containing 500 μl of sterile 50% glycerol. Samples were kept on wet ice and transported to the laboratory. Upon arrival samples were thoroughly vortexed and plated (100 μl for the nasopharyngeal sample and 50 μl for the oropharyngeal and saliva samples) onto blood agar plates supplemented with 5 µg/mL gentamicin, and grown overnight in anaerobic jars at 37 °C. The total bacterial growth was collected and frozen at −80 °C in STGG medium. From these tubes DNA was extracted and tested as described below.

### Isolation of single colonies from polymicrobial sample

Serial dilutions up to 10^−8^ were performed for one polymicrobial sample from saliva. Fifty microliters of each dilution were inoculated onto blood agar plates supplemented with 5 µg/mL gentamicin, and incubated overnight at 37 °C in 5% CO_2_. On the following day plates were inspected for the presence of single colonies and a total of 600 colonies were picked randomly. Each colony was streaked in a 1/10 slice of a novel blood agar plate. The plates obtained (n = 60) were incubated as above. On the following day, the 10 cultures in each plate were collected from a pre-defined circle area in the center of the plate. This ensured that similar amounts of each culture were collected. Pools of 10 cultures were resuspended in PBS. These pools were used for DNA extraction.

### Genomic DNA extraction

DNA was extracted from pure cultures using the MagNa Pure Compact Nucleic Acid Isolation kit (Roche Diagnostics GmbH) or the DNeasy Blood & Tissue kit (Qiagen) as recommended by the manufacturers. DNA from polymicrobial samples was extracted using the MagNa Pure Compact Nucleic Acid Isolation kit. To control for DNA contamination, during DNA extraction, for each batch of seven samples being processed, one additional sample containing only ultrapure water was processed in parallel. DNA quantification and purity were evaluated with NanoDrop (Thermo Fisher). DNA for whole genome sequencing was treated with RNase and analyzed with NanoDrop and Qubit (Thermo Fisher).

### Design of real-time PCR assay targeting SP2020

SP2020 (encoding for a putative transcriptional regulator) was initially selected by *in silico* screening of 27 genomes of *S. pneumoniae* for genes that are highly conserved, universally present and specific to the species. Upon identification of SP2020 as a potential candidate, BLAST analysis was conducted against the NCBI database using the same criteria. In addition, SP2020 has been evaluated with ~15,000 samples as one of the control genes for pneumococcus in the BμG@S SP-CPS microarray (J. Hinds, personal communication).

To design the real-time PCR assay targeting SP2020, the nucleotide sequence of the TIGR4 SP2020 gene (NCBI accession number AE 005672.3, nt 1925563 to 1926291) was blasted against the NCBI database (as of November 2015). Homology was found only to pneumococcal nucleotide sequences (29 sequences, 99–100% nucleotide similarity) and not to any other *Streptococcus* species. One set of primers and a FAM-labeled probe were custom-designed (Metabion International AG):

SP_2020_F (5′-TAAACAGTTTGCCTGTAGTCG-3′),

SP_2020_R (5′-CCCGGATATCTCTTTCTGGA-3′), and

SP_2020_P (5′-Fam-AACCTTTGTTCTCTCTCGTGGCAGCTCAA-BHQ-3′). This combination of primers resulted in an amplicon length of 155 bp (nt 1926036 to 1926190 of TIGR4). The real-time PCR assay was tested and optimized for *S. pneumoniae* TIGR4 and *S. pseudopneumoniae* ATCC BAA-960 (NCBI accession number AM113495.1).

### Real-time PCR targeting *lytA*, *piaB*, and SP2020

Assays were performed according to the MIQE guidelines^39^. The presence of the genes *lytA* and *piaB* was tested by using primers and probes previously described^7,11^. The presence of SP2020 was tested by using primers and probes described above. For DNA obtained from pure cultures 2.5 μL of DNA at 0.2 ng/μL were used in each reaction. For polymicrobial samples 2.5 μL of DNA were used regardless of their concentration. All reactions were performed in a final volume of 25 μL containing 1x FastStart TaqMan Probe Master (Roche), 0.15 mM each primer, 0.075 mM probe. DNA was amplified with the CFX96 Real-Time System Amplification (Bio-Rad) by using the following cycling conditions: 95 °C for 10 min followed by 45 cycles of 95 °C for 15 sec, 60 °C (for *lytA*-CDC and *piaB*) or 55 °C (for SP2020) for 1 min. Fluorescence was read after each of the 45 cycles.

All strains from *S. pneumoniae* control collection and non-*S. pneumoniae Streptococcus spp*. control collection were tested twice on different days. All strains from the α-hemolytic non-pneumococcal streptococcal collection were tested once, except when amplification occurred. In such cases, isolates were re-tested on a different day for confirmation. In addition, for each assay, a random selection of 10% of the strains from the test collection was also independently selected for re-testing. DNA from *S. pneumoniae* TIGR4 (positive control) and *S. pseudopneumoniae* ATCC BAA-960 (negative control) were used in every run. Samples were considered positive when the cycle threshold (C_T_) value was equal or below 40.

Similarly, polymicrobial samples were tested once, except when amplification occurred. In such cases, isolates were re-tested on a different day for confirmation.

Contamination assessment during real-time PCR assays was evaluated systematically in all runs by testing extracted ultrapure water (described above), water used in DNA dilutions, and water used in the real-time PCR reactions.

In addition, all non-pneumoccoccal streptococcal isolates giving positive results for the real-time PCR assays were further evaluated: single colonies were picked from each culture and streaked for five consecutive days to confirm sample purity. On the fifth day, a new culture stock was done, DNA was extracted, real-time PCR assays were repeated and the results were confirmed. Whole genome sequencing analysis was performed as described below.

### Performance of real-time PCR assays for the identification of pneumococcus

To evaluate the performance of the real-time PCR assays for the identification of pneumococcus, four parameters were estimated: sensitivity (to estimate the percentage of pneumococci correctly identified among all pneumococci tested), specificity (to estimate the percentage of non-pneumococci correctly identified among all non-pneumococci tested), positive predictive value (PPV, to estimate the percentage of pneumococci among all isolates giving a positive result for a given assay), and negative predictive value (NPV, to estimate the percentage of non-pneumococci among all isolates giving a negative result for a given assay). Sensitivities and specificities of real-time PCR assays were compared using the McNemar test^40^. PPVs and NPVs of real-time PCR assays were compared using the generalized score statistic proposed by Leisenring *et al*.^41^. Both statistics were calculated using the R package DTComPair^42^.

### Real-time PCR serotyping

All polymicrobial samples giving results suggesting the presence of pneumococci were further tested using a panel of primers and probes for serotype assignment (1, 2, 3, 4, 5, 6A/B/C/D, 7A/F, 9A/V, 11A/D, 12A/B/F/44/46, 14, 15A/F, 16F, 18A/B/C/F, 19A, 19F, 22A/F, 23A, 23F, 33A/F/37^43^, 8, 10A/B and 38)^44^ as previously described^45^.

### Whole genome sequencing and genomic comparison with TIGR4

Genomes of five non-pneumococcal isolates testing positive for at least one of the real-time PCR assays and of one capsulated pneumococcal isolate giving a negative result for *piaB* were sequenced by the Illumina MiSeq platform, with a minimum coverage of 100x. Library preparation and sequencing were done at the Genomics Unit of Instituto Gulbenkian de Ciência (Oeiras, Portugal). Paired-end reads were checked for quality, trimmed, and de novo assembled using the Qiagen CLC Genomics Workbench version 9.0.1 software (Qiagen, Venlo, The Netherlands). Bubble size, word size and paired distances were automatically calculated by the software. The consensus sequences were extracted and the contigs were deposited in NCBI database and annotated with the Prokaryotic Genomic Annotation Pipeline. Read data, assembled and annotated contigs of the six sequenced isolates were deposited in the NCBI database: BioProject accession number PRJNA434586.

For the pneumococcal isolate, the assembled contigs were ordered against TIGR4 complete genome using Mauve version 2.3.1^46^ and concatenated using Artemis release 17.0.1^47^. Genomic comparison between TIGR4 and the pneumococcal isolate was performed using Double ACT v2 and visualized using Artemis Comparison Tool (ACT)^48^. For the non-pneumococcal isolates, assembled contigs were blasted against the nucleotide sequences of TIGR4 *lytA* (SP_1937, nt 1840405 to 1841361), TIGR4 *piaB* (SP_1033, nt 974409 to 975428) or TIGR4 SP2020 (SP_2020, nt 1925563 to 1926291). Comparisons between TIGR4 complete genome and the hit contigs were performed by Double ACT v2 and visualized using Artemis Comparison Tool (ACT).

### Multilocus sequence analysis (MLSA) for viridans group streptococci (viridans MLSA)

DNA sequences of the seven housekeeping genes *map*, *pfl*, *ppaC*, *pyk*, *rpoB*, *sodA*, and *tuf* were extracted from whole genome sequencing data. Phylogenetic analysis of the concatenated sequences in comparison with the eMLSA database (http://www.emlsa.net/) was performed using MEGA6.06 (http://www.megasoftware.net) as described: alignment of sequences by ClustalW and construction of a minimum-evolution phylogenetic tree using default parameters^49,50^.

### Multilocus sequence typing (MLST) using the *S. pneumoniae* MLST database

Allele assignment for the seven housekeeping genes *aroE*, *ddl, gdh, gki*, *recP*, *spi*, and *xpt* was inferred from whole genome sequencing data using the tool MLST 1.8 provided by the Center for Genomic Epidemiology^51^.

### *In silico* screening for *lytA, piaB*, and SP2020 of pneumococcal genomes

An *in silico* analyses to screen for the absence of *lytA, piaB*, or SP2020 was performed for 8251 pneumococcal genomes available at NCBI database (https://www.ncbi.nlm.nih.gov/genome/?term=streptococcus+pneumoniae, accessed on July 9, 2018). Sequences were extracted as fasta files and imported into Qiagen CLC Genomics Workbench version 9.0.1 software. Blasts were performed against the nucleotide sequences of TIGR4 *lytA* (SP_1937, nt 1840405 to 1841361), TIGR4 *piaB* (SP_1033, nt 974409 to 975428) and TIGR4 SP2020 (SP_2020, nt 1925563 to 1926291).

### Ethics statement

The study was conducted in accordance with the European Statements for Good Clinical Practice and the declaration of Helsinki of the World Health Medical Association and is integrated in a project approved by “Conselho de Ética para a Saúde do Instituto de Higiene e Medicina Tropical da Universidade Nova de Lisboa” (Process No. 04-2014-PN). Written, informed consent was obtained from all participants providing biological samples.

## Supplementary information

Supplementary Tables
