# Supplementary material for: Identification of Streptococcus pneumoniae by a real-time PCR assay targeting SP2020
Source: Sci Rep. 2019 Mar 1;9:3285. doi: 10.1038/s41598-019-39791-1 (PMC6397248; doi:10.1038/s41598-019-39791-1)
Supplement: Supplementary file 1 — Supplementary Tables [file 41598_2019_39791_MOESM1_ESM.pdf]

Identification of *Streptococcus pneumoniae* by a real-time PCR assay targeting SP2020

Débora A. Tavares[1§], Sara Handem[1§], Ricardo J. Carvalho[1], A. Cristina Paulo[1], Hermínia de Lencastre[2,3], Jason Hinds[4,], and Raquel Sá-Leão[1,5*]

Supplementary Table 1. Comparison of performance of three real-time PCR assays.

Assay	Sensitivity		Specificity		PPV		NPV	
	%	p-value	%	p-value	%	p-value	%	p-value
lytA vs *piaB*	100 vs 95.3	**0.0082**	99.5 vs 99.5	1.0000	98.7 vs 98.6	0.9620	100 vs 98.4	**0.0082**
lytA vs SP2020	100 vs 100	1.0000	99.5 vs 99.8	0.5640	98.7 vs 99.3	0.5633	100 vs 100	1.0000
piaB vs SP2020	95.3 vs 100	**0.0082**	99.5 vs 99.8	0.5640	98.6 vs 99.3	0.5390	98.4 vs 100	**0.0082**
lytA+*piaB* vs *lytA*+SP2020	95.3 vs 100	**0.0082**	100 vs 100	1.0000	100 vs 100	1.0000	98.4 vs 100	**0.0082**
piaB+SP2020 vs *lytA*+SP2020	95.3 vs 100	**0.0082**	100 vs 100	1.0000	100 vs 100	1.0000	98.4 vs 100	**0.0082**
lytA vs l*ytA*+SP2020	100 vs 100	1.0000	99.5 vs 100	0.1570	98.7 vs 100	0.1550	100 vs 100	1.0000
SP2020 vs *lytA*+SP2020	100 vs 100	1.0000	99.8 vs 100	0.1570	99.3 vs 100	0.1550	100 vs 100	1.0000

Statistical analysis was performed based on McNemar test and generalized score statistic. P-values<0.05 are highlighted in bold.

Supplementary Table 2. *S. pneumoniae* strains lacking *lytA*, *piaB*, or SP2020 based on an *in silico* screening of 8209 genomes publicly available at NCBI (accessed on August 9, 2018).

NCBI Reference Sequence	Serotype[1]	MLST	Observation
Genomes lacking *lytA* (n=1)			
NZ_MQRO01000001.1[2]	3	505	
Genomes lacking *piaB* (n=250)			
203 NT genomes	NT	several	
NZ_CLDL01000001.1	6A	5477	
NZ_CGFX01000001.1	6B	90	
NZ_CHCX01000001.1	6B	90	
NZ_CHDJ01000001.1	6B	90	
NZ_CHYS01000001.1	6B	90[3]	
NZ_CPKV01000001.1	6B	90	
NZ_CPLC01000001.1	6B	90	
NZ_CPLR01000001.1	6B	90	
NZ_CPMF01000001.1	6B	90	
NZ_CPMJ01000001.1	6B	90	
NZ_CPMZ01000001.1	6B	90	
NZ_CPNF01000001.1	6B	90	
NZ_CPNK01000001.1	6B	90	
NZ_CPNL01000001.1	6B	90	
NZ_CPON01000001.1	6B	90	
NZ_CPQM01000001.1	6B	90	
NZ_CPQV01000001.1	6B	90	
NZ_CPRD01000001.1	6B	90	
NZ_CPRZ01000001.1	6B	90	
NZ_CPSW01000001.1	6B	90	
NZ_CPTP01000001.1	6B	90	
NZ_CPTW01000001.1	6B	90	
NZ_CPTX01000001.1	6B	90	
NZ_CPUS01000001.1	6B	90	
NZ_CRXR01000001.1	6B	90	
NZ_CVIP01000001.1	6B	90	
NZ_AFGA01000011.1	6B	9057	SLV of ST1536
NZ_AGPE01000012.1	6B	1536	QLV of ST90
NZ_FYFF01000075.1	6B	2457	QLV of ST1536
NZ_FWSR01000314.1	6B	384	TLV of ST1536
NZ_FFRF01000001.1	9N	4666	
NZ_FZEP01000080.1	10A	449	
NZ_CP018838.1	11A	8279	
NZ_FFRD01000001.1	12F	3776	
NZ_FZDR01000051.1	15B	448	
NZ_CHYT01000001.1	17F/3	392	
NZ_CPOL01000001.1	17F/4	392	
NZ_CFQP02000001.1	19A	276	
NZ_CMOT01000001.1	19A	230	SLV of ST276
NZ_CGZC01000001.1	19A	1701	DLV of ST276
NZ_FHGX01000001.1	19A	292	
NZ_MDYD01000001.1	19A	2062	
NZ_AFAX01000009.1	19F	236	
NZ_CPLH01000001.1	19F	4414	SLV of ST236

NZ_CRVH01000001.1	19F	4414	SLV of ST236
NZ_COEP02000001.1	35B	558	
NZ_CVLR01000001.1	35F	4418	
Genomes lacking SP2020 (n=23)			
NZ_CHGO01000001.1	NT	5107	
NZ_CHVY01000001.1	NT	5107	
NZ_CKGU01000001.1	NT	5107	
NZ_CKRN01000001.1	NT	5107	
NZ_CMAT01000001.1	NT	5107	
NZ_CJZH01000001.1	NT	6687	
NZ_CKLF01000001.1	NT	6687[4]	
NZ_CJZJ01000001.1	NT	4131	
NZ_CKTF01000001.1	NT	13122	
NZ_CMTI01000001.1	NT	13081	
NZ_MQRU01000001.1	3	several ST possible[5]	
NZ_ALCD01000001.1	7C	1207 or 1797[6]	
NZ_CFIQ02000001.1	19F	425	
NZ_CNQY02000001.1	19F	425	
NZ_CNQZ02000001.1	19F	425	
NZ_CNRV02000001.1	19F	425	
NZ_CNTY02000001.1	19F	425	
NZ_CNUL02000001.1	19F	425	
NZ_CP025076.1	19F	425	
NZ_CNOX02000001.1	19F	1903	SLV of ST425
NZ_FHQJ01000001.1	33F	2705	
NZ_FHQU01000001.1	33F	2705	
NZ_FHRE01000001.1	33F	2705	
Genomes lacking *piaB* and SP2020			
NZ_LTCN01000001.1	NT	11884	

[1]ND, serotype not determined. [2]A large rearrangement resulting in the deletion of a region of 79.6 kb that includes *lytA* seems to have occurred. [3]*gki* allele is missing; the closest ST is indicated. [4]*xpt* allelle is missing; the closest ST is indicated. [5]*recP* allele is missing; 19 STs share the six other alleles. [6]*recP* allele is missing; the closest ST is indicated. *S. pneumoniae* MLST database was last accessed on September 11, 2018. SLV, DLV, TLV and QLV indicate single, double, triple and quadruple locus variants, respectively.

Supplementary Table 3. C_T values obtained for polymicrobial samples tested by real-time PCR for the presence of *lytA*, *piaB* and SP2020. A total of eighty samples were tested for each sampling site. Only positive results are shown. NA, not available.

Samples	Real-time PCR (C_T)		
	lytA	***piaB***	**SP2020**
**Nasopharynx**			
18	18	18	16
32	24	24	22
28	19	20	19
36	26	26	28
46	20	18	19
50	17	17	18
61	21	NA	NA
72	16	15	17
78	17	NA	35
100	22	21	32
118	24	25	29
129	15	15	14
132	18	NA	43
137	18	18	18
141	22	22	24
157	19	19	19
172	24	23	23
175	18	NA	NA
176	19	18	18
190	18	NA	43
192	19	19	18
205	20	19	16
211	21	21	17
216	26	26	23
233	22	23	25,NA
323	20	20	22
367	NA	NA	34
427	14	NA	35
455	19	20	18
463	18	18	16
547	23	23	23
364	18	18	16
**Oropharynx**			
21	27	27	28
23	25	26	28
30	25	26	28
36	26	26	36
46	28	27	29
72	21	21	24
74	27	27	32
80	23	23	25
87	25	25	25
88	32	31	30

152	26	26	26
172	36	35	39
187	NA	NA	33
194	NA	NA	38
216	29	29	28
323	27	28	36
389	32	32	33
411	NA	NA	35
478	23	24	23
507	28	29	31
609	29	29	30
**Saliva**			
15	30	31	31
36	NA	NA	39
47	NA	NA	34
106	NA	NA	34
118	34	34	34
133	NA	NA	38
170	31	32	34
250	33	33	29
280	NA	NA	35
283	27	27	26
292	NA	NA	38
332	NA	NA	34
353	NA	NA	37
366	NA	NA	31
406	NA	NA	38
415	35	36	36
421	NA	NA	38
440	NA	NA	35
469	NA	NA	34
478	30	30	30
493	37	37	41
531	23	24	23
538	NA	NA	35
559	NA	NA	37
563	NA	NA	32
564	32	32	33
579	23	24	24
